# Effects of pH on the Properties of Membrane Vesicles Including Glucosyltransferase in *Streptococcus mutans*

**DOI:** 10.3390/microorganisms9112308

**Published:** 2021-11-06

**Authors:** Yusuke Iwabuchi, Tomoyo Nakamura, Yasuka Kusumoto, Ryoma Nakao, Tsutomu Iwamoto, Osamu Shinozuka, Hidenobu Senpuku

**Affiliations:** 1 Department of Pediatric Dentistry/Special Needs Dentistry, Graduate School of Medical and Dental Sciences, Tokyo Medical and Dental University, Tokyo 113-8519, Japan; iwbcdpd@tmd.ac.jp (Y.I.); yasudysf@tmd.ac.jp (Y.K.); iwamoto.dohs@tmd.ac.jp (T.I.); o.shinozuka.dpd@tmd.ac.jp (O.S.); 2 Department of Bacteriology I, National Institute of Infectious Diseases, Tokyo 162-8640, Japan; nakamuraty@stf.teu.ac.jp (T.N.); ryoma73@nih.go.jp (R.N.); 3 School of Bioscience and Biotechnology, Tokyo University of Technology, Tokyo 192-0982, Japan; 4 Department of Microbiology and Immunology, Nihon University School of Dentistry at Matsudo, Chiba 271-8587, Japan

**Keywords:** biofilm, membrane vesicle, GtfC, initial pH, acetic acid

## Abstract

*Streptococcus mutans* releases membrane vesicles (MVs) and induces MV-dependent biofilm formation. Glucosyltransferases (Gtfs) are bound to MVs and contribute to the adhesion and glucans-dependent biofilm formation of early adherent bacteria on the tooth surface. The biofilm formation of *S. mutans* may be controlled depending on whether the initial pH tends to be acidic or alkaline. In this study, the characteristics and effects of MVs extracted from various conditions {(initial pH 6.0 and 8.0 media prepared with lactic acid (LA) and acetic acid (AA), and with NaOH (NO), respectively)} on the biofilm formation of *S. mutans* and early adherent bacteria were investigated. The quantitative changes in glucans between primary pH 6.0 and 8.0 conditions were observed, associated with different activities affecting MV-dependent biofilm formation. The decreased amount of Gtfs on MVs under the initial pH 6.0 conditions strongly guided low levels of MV-dependent biofilm formation. However, in the initial pH 6.0 and 8.0 solutions prepared with AA and NO, the MVs in the biofilm appeared to be formed by the expression of glucans and/or extracellular DNA. These results suggest that the environmental pH conditions established by acid and alkaline factors determine the differences in the local pathogenic activities of biofilm development in the oral cavity.

## 1. Introduction

*Streptococcus mutans* is an oral bacterium that is importantly involved in the development of dental caries and is a principal inducer of the oral biofilm formation containing various bacteria on the tooth surface [1,2,3,4]. *S. mutans* is capable of releasing three types of glucosyltransferases (Gtfs, i.e., GtfB, GtfC, and GtfD) and synthesizing water-insoluble glucan and water-soluble glucan from sucrose by GtfB and GtfC to form a sticky oral biofilm [1,2,4,5]. The aggregation of bacterial cells using water-insoluble glucan synthesized by GtfB and the adhesion of bacteria using water-soluble and water-insoluble glucan synthesized by GtfC on the tooth surface are important aspects for the formation of biofilms, including other bacteria [4,5].

In recent years, it has been clearly reported that not only Gram-negative bacteria, but also Gram-positive bacteria such as *S. mutans* release membrane vesicles (MVs) [6,7,8,9,10,11]. MVs contain many factors such as lipopolysaccharide (LPS), lipoprotein, peptidoglycan, enzymes, and virulence-associated factors [12,13,14]. Furthermore, MVs are highly involved in the development of biofilm formation [11,15]. A relationship has also been found between biofilm formation and the MVs from pathogens such as the *Francisella* species, *Pseudomonas aeruginosa*, *Vibrio cholerae*, and *Helicobacter pylori* [16,17,18]. The MVs from *S. mutans* contain Gtfs, bacterial cell surface protein antigen (PAc), and a glucan-binding protein, which are components of *S. mutans* associated with the development of dental caries [10,11,19]. Among Gtfs, GtfC is mainly bound to MVs, and the MVs are used for the adhesion and biofilm formation of other early adherent bacteria and core bacteria in oral biofilms on the tooth surface, such as *Streptococcus mitis, Streptococcus oralis, Actinomyces naeslundii,* and *Fusobacterium nucleatum* [11,19]. The MVs of *S. mutans* bind to the acquired pellicle on the tooth surface by the ability of PAc, and as a result, GtfC may be guided to the tooth surface; subsequently, GtfC synthesizes water-soluble glucan and water-insoluble glucan on the tooth surface in a sugar-containing environment [19,20,21,22]. It has been suggested that MVs utilize water-soluble glucans and water-insoluble glucans to support the binding of early adherent bacteria to the tooth surface [11]. After the biofilm is formed on the tooth surface, short-chain fatty acids such as lactic acid and acetic acid are produced in and around the biofilm to lower the pH [23,24,25,26]. This decrease in pH can affect subsequent biofilm formation [23]. If the quantity of bacteria increases on the tooth surface, the pH will drop further when sugars are present, and the bacteria will experience stress at a lower pH. However, acid-resistant bacteria such as *S. mutans* have several acid-resistant genes including *ffh, uvrA*, and *dnaK* [27]. In an attempt to prevent acid-induced cell death, the bacteria are stimulated by the lowered pH, causing various responses [28,29,30]. In a more recent paper, *S. mutans* produced MVs under acid pressure to carry proteins associated with cariogenesis [31]. The alterations of *S. mutans* MVs were associated with ABC transporters. These factors may lead to a survival response with MVs or reduced death in bacteria, such as proton pumping and bacteriocin production in quorum sensing [32,33,34,35]. The shift of pH to the alkaline side due to gingival crevicular fluid may also affect subgingival biofilm formation. The concentration of formic acid produced after the fermentation of sugars by oral bacteria in the gingival crevicular fluid has an inverse relationship with the severity of periodontitis [36]. This observation may reflect the phenomenon that plaques tend to accumulate and associate with periodontitis under the subgingival area. In one study, the properties of the biofilm formed when the biofilm formation experiment was conducted at an initial pH of 6.0 were clearly different from those of the biofilm formed at pH 7.0 [37]. Therefore, different properties in biofilm formation are observed when the biofilm adheres and remains on the tooth surface to produce acid, lowering the pH, and for subgingival biofilm under the influence of gingival crevicular fluid. The effect of the initial pH on biofilm formation activity, including MVs formation, has not been clearly studied, and the characteristics of the MVs under the various pH conditions may affect subsequent biofilm formation.

To clarify the effects of the initial pH on the MVs formation, this study assesses the effects on the production of MVs when a *S. mutans* culture is performed with the pH lowered to 6.0 by lactic acid and acetic acid, produced by oral bacteria, and whether MV-dependent biofilm formation is possible. Furthermore, this study examines the kinds of MVs that are formed to induce biofilm formation when the solution is adjusted with NaOH to an alkaline value of pH 8.0. As a result, a new interpretation is possible for pH-controlled MV-dependent biofilm formation. This research may contribute to the development of biofilm formation inhibitors that target MVs.

## 2. Materials and Methods

### 2.1. Bacterial Strains and Culture Conditions

The bacterial strains *S. mutans* UA159 [38], *S. mutans* UA159 *gtfBC* mutant (*gtfBC^-^*) [39], *S. mutans* UA159 *SMU833* mutant (*SMU833^-^*) [40], *S. mutans* UA159 *sacB* mutant (*sacB^-^*) [41], *Streptococcus mitis* ATCC 903, and *Actinomyces naeslundii* x600 were maintained and cultured in a brain-heart infusion (BHI) broth (Becton Dickinson and Company, Franklin Lakes, NJ, USA) at 37 °C in an atmosphere containing 5% CO_2_ (Gas pack: Mitsubishi Gas Chemical Co., Inc., Tokyo, Japan). The prepared cells were used in this study.

### 2.2. Human Saliva Collection

Whole saliva samples stimulated by the chewing of paraffin gum were collected from 3 healthy human volunteers (25–29 years old) and pooled into ice-chilled sterile bottles over a period of 5 min. The samples were clarified by centrifugation at 10,000× *g* for 10 min at 4 °C, sterilized using 0.22-µm and 0.45-µm Millex-GP Filter Units (Merck kGaA), and a 20 mL saliva sample was added into each well in 96-well polystyrene microtiter plates (Sumitomo Bakelite, Tokyo, Japan) to coat salivary components for biofilm formation assays. 

### 2.3. Extraction of MVs

*S. mutans* was cultivated in 1000 mL of BHI broth (control, pH 7.4); BHI broth, pH 6.0, prepared with HCl (HC), lactic acid (LA) and acetic acid (AA), separately; and BHI broth, pH 8.0, prepared with NaOH (NO) at 37 °C overnight in an atmosphere containing 5% CO_2_. The initial cell density was 1.5 × 10^8^, and the final cell density was 1.5 × 10^11^ in 1000 mL. The preparation of MVs was performed as described previously with some modifications [11]. Culture supernatants were separated by centrifugation (6000× *g* for 20 min) and concentrated to >50 kDa by centrifugal filtration (Amicon Ultra 4, Merck kGaA, Darmstadt, Germany or VIVASPIN 20, Sartorius, Stone House, United Kingdom). Briefly, the concentrated MVs were filtered through polyvinylidene difluoride (PVDF) filter membranes (Merck kGaA) with pore sizes of 0.45 and 0.22 μm. The MVs samples were centrifuged (150,000× *g* for 2 h) by a Beckman SW 41 Ti swinging bucket rotor using a Beckman optima L-90k ultracentrifuge (Beckman Coulter, South Kraemer Boulevard, CA, USA), and the pellets were suspended in sterile phosphate-buffered saline (PBS; pH 7.2). The samples were also ultracentrifuged (150,000× g at 4 °C for 2 h), and the pellets were resuspended in 200 μL of sterile PBS as the sample. The suspended samples were called MVs and were used for the following experiments. The MVs protein concentration was determined using a Bio-Rad Protein Assay kit (Bio-Rad Laboratories, Inc., Hercules, CA, USA).

### 2.4. Scanning Electron Microscopy (SEM)

For the analysis of MVs derived from *S. mutans* and *S. mutans* cultivated in 1000 mL of BHI broth (control, HC, LA and AA, and NO), MVs were collected from culture supernatants by centrifugation at 150,000× g and washed twice with PBS by centrifugation at 150,000× g. Each MVs sample was set to 150 ng/µL protein in PBS, and each strain of bacteria was set to an optical density at 600 nm (OD600) of 0.6 in PBS. Both samples were mixed with equal amounts of 2.5% glutaraldehyde and 2% paraformaldehyde in PBS. The samples were washed in PBS, dehydrated in a graded ethanol series ranging from 50% ethanol to absolute ethanol, immersed in isoamyl acetate, dried by critical point drying, coated with osmium vapor using an osmium plasma coater, and visualized by scanning electron microscopy (SEM, S-5200, HITACHI, Hitachi, Japan). The areas of the MVs were measured by Image-Pro (Media Cybernetics, Rockville, MD).

### 2.5. Biofilm Formation Assay

Biofilms from each strain were developed in 96-well polystyrene microtiter plates (Sumitomo Bakelite), which were previously coated with human saliva. Biofilm formation assays were performed using a previous procedure [42]. Overnight cultures of *S. mutans* UA159.*gtfBC*^-^ and other bacteria (*S. mitis* ATCC 903 and *A. naeslundii* x600) in BHI broth were inoculated at a ratio of 1:100 in 200 µL of tryptic soy broth (TSB, Becton Dickinson and Company) with 0.25% sucrose or 0.25% raffinose with and without various concentrations of MVs. To observe the contribution of extracellular DNA (eDNA) in the MVs-dependent biofilm formation, 50 units/ ml DNase I (Roche Applied Science, Mannheim, Germany) and DNase I heated by boiling for 10 min (iDNase I) were added in the biofilm formation assay. The plates were incubated at 37 °C in 5% CO_2_ aerobic conditions for 16 h. After incubation, the planktonic cells were removed by washing with distilled water (DW), and the adherent cells were stained with 0.25% safranin for 15 min to determine the biofilm formation level. After washing with DW 2 times, safranin was extracted from the biofilms with 70% (vol/vol) ethanol. The biofilm formation was quantified by measuring the absorbance of the stained biofilms at 492 nm.

### 2.6. Observation of Live Cells and Glucan in Biofilm Formation

Biofilms were stained by the FilmTracer *Live/Dead* Biofilm Viability Kit (Molecular Probes, Inc., Eugene, OR, USA), where SYTO 9 was added to the biofilms at a final concentration of 5 µM. Before the incubation, Alexa Fluor 647-dextran conjugate (Molecular Probes, Invitrogen Corp., Carlsbad, CA, USA) [4] was added into the bacteria suspension in the wells of a 96 microtiter plate and incubated for red fluorescence at 37 °C in 5% CO_2_ aerobic conditions for 16 h, while the nucleic acids in the bacterial cells were labeled with SYTO 9 to produce green fluorescence.

The biofilms were incubated with the dyes at room temperature for 20–40 min before being imaged by an LSM700 Meta NLO confocal laser scanning microscopy (CLSM) system (Carl Zeiss Inc., Jena, Germany). The biofilms were observed by CLSM, and two-dimensional (2D) images were acquired by a Plan-Apochromat 10 x/0.45 M27 lens objective. Confocal images for biofilm formation were visually observed by ZEN (Carl Zeiss) analysis software.

### 2.7. SDS-PAGE and Western Blotting

Prior to electrophoretic analysis, the MVs were diluted with an equal volume of SDS-PAGE buffer [0.06 M Tris-HCl (Amersham Pharmacia Biotech, Buckinghamshire, UK), pH 6.8, 20% glycerol (Wako Pure Chemical Industries Ltd., Osaka, Japan), 0 or 1% (wt/vol) SDS (Wako), 1% 2-ME, and 0.0012% bromophenol blue (Wako)]. The SDS-PAGE samples were heated at 100 °C for 5 min just prior to loading on the gel. The SDS-PAGE samples were then applied on a 6.0% or 12.5% polyacrylamide gel (e-PAGEL, ATTO Corp., Tokyo, Japan) in 0.025 M Tris-HCl, 192 mM glycine (Wako) and 0 or 0.1% (wt/vol) SDS. Electrophoretic separation of the proteins was carried out for 70 min at 25 mA. Gels were stained with Coomassie Brilliant Blue (CBB, Wako) and ethidium bromide to observe the proteins and DNA, respectively.

The MVs were lysed by adding an equal volume of 1× SDS sample buffer [2% SDS, 50 mM Tris-HCl (pH 6.8), 10% glycerol, 2.5% 2-ME, 0.1% bromophenol blue] and boiling for 5 min at 95 °C. Equal amounts of each protein sample were then separated by 6.0% or 12.5% acrylamide SDS-PAGE. Subsequently, proteins were transferred onto Immobilon PVDF membranes (Millipore, Bedford, MA, USA) and blocked with 2.5% skim milk in Tris-buffered saline with Tween 20 (TBST: 50 mM Tris, 2.7 mM KCl, 0.138 M NaCl, 0.05% Tween 20, pH 7.6) for 1 h at room temperature. The membranes were probed with mouse-derived anti-MV antisera [19] diluted 1:15,000 in 1.25% skim milk/TBST for 1 h at room temperature. Horseradish peroxidase (HRP)-labeled anti-goat secondary antibody (Merck kGaA) was used to detect the antibodies. Optical emission signals on the proteins were produced by enhanced chemiluminescence (ECL Western blotting Substrate, Thermo Scientific, Southfield, MI) and detected with a FUSION SOLO.7S. EDGE system (Vilber Lourmat, Marne-la-Vallée, France).

### 2.8. Zymography

Zymography was performed using sucrose or raffinose-containing gels as described by Mattos-Graner et al. [43]. First, 2 µg of MVs was suspended in an equal volume of sodium dodecyl sulfate polyacrylamide gel electrophoresis (SDS-PAGE) buffer without 2-mercaptoethanol (2-ME, Merk kGaA) and heated at 100 °C for 5 min. The samples were loaded into 6% or 12.5% polyacrylamide gel, SDS-PAGE was performed, and the gels were washed twice for 15 min with zymogram lysis buffer (2.5% Triton X-100) and then incubated at 37 °C overnight or 3 days in a reaction buffer [0.2 M sucrose in 0.2 M phosphate buffer (pH 7.3) or 0.125 M raffinose in 0.05 M acetate buffer (pH 5.5)]. The Gtf and fructosyltransferase (Ftf) activities were identified as white bands, and the gel was photographed with a black background.

### 2.9. Peptide Mass Fingerprinting (PMF) Analyses of MVs

First, 2 μg of MVs was subjected to 6% polyacrylamide SDS-PAGE and CBB staining, and prominent protein bands (at approximately 90 kDa and 50 kDa) were selected and submitted to GENOMINE Inc. (Pohang, Korea) for PMF analysis using matrix-assisted laser desorption/ionization time-of-flight (MALDI-TOF) mass spectrometry.

### 2.10. Total RNA Extraction and Real-Time PCR

*S. mutans* UA159 was cultivated in 1.5 mL of the control, HC, LA, AA, and NO group solutions. The samples were incubated at 37 °C in 5% CO_2_ under aerobic conditions for 6 h. The total RNA was extracted using ISOGEN (Nippon Gene, Tokyo, Japan) according to the manufacturer’s method for extracting RNA from Gram-positive bacteria, and was then suspended in 100 µL of RNase-free water. The extracted total RNA was cleaned up by an RNeasy Mini Kit (Qiagen, Hilden, Germany). Next, 0.5 µg of total RNA was reverse-transcribed using the ReverTra Ace qPCR RT master mix with gDNA remover (Toyobo, Osaka, Japan) to obtain complementary DNA (cDNA).

As a negative control, a sample without reverse transcriptase was also prepared to confirm that the signal in the following quantitative RT-PCR was derived from cDNA. A quantitative reverse transcription PCR (RT-qPCR) was performed on an ABI Prism 7000 instrument (Applied Biosystems, CA, USA) using Power SYBR Green PCR master mix (Applied Biosystems, Warrington, UK) as previously described [43]. The expression levels were determined by the relative quantitative comparative threshold cycling method (ΔΔCT). The cycling conditions were as follows: initial denaturation cycling conditions of 2 min at 95 °C, 40 cycles of 15 s at 95 °C, and 40 s at 60 °C. The expression levels were normalized to lactate dehydrogenase mRNA (endogenous control). The primers used for qPCR are shown in Table 1.

### 2.11. Statistical Analysis

The biofilm formation levels were expressed as the mean ± standard deviation. In the biofilm assay, the statistical significance of differences between the bacteria with and without various concentrations of the MVs was determined using a one-way analysis of variance (ANOVA) followed with Bonferroni correction (IBM SPSS statistics 24, IBM Corporation, Armonk, NY, USA). A *p*-value less than 0.05 was considered statistically significant. All experiments were repeated independently three times.

## 3. Results

### 3.1. Effects of Initial pH on the Formation of MVs in Growth Condition

**Figure 1:**
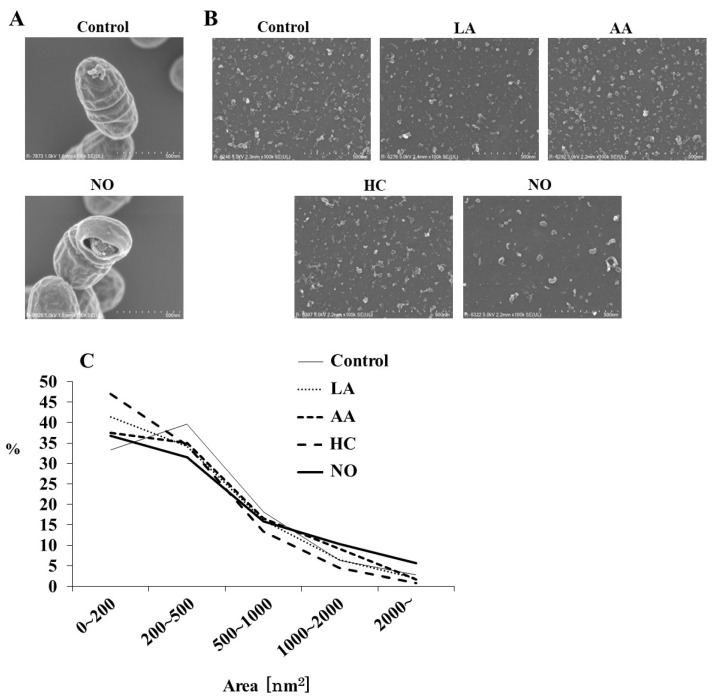
SEM analysis of *S. mutans* MVs. *S. mutans* UA159 with MVs in control and initial pH 8.0 condition prepared with NO were observed at magnifications of 100,000 by SEM (**A**), and MVs in control, initial pH 6.0 conditions prepared with LA, AA, and HC, and initial pH 8.0 prepared with NO were observed at 100,000 (**B**). Representative data from more than three independent experiments are shown in the pictures. The size (area) of MVs was also calculated by SEM (**C**).

To observe the effects of the initial pH on the formation of MVs from *S. mutans*, media using a BHI broth were separately prepared at pH 6.0 by HC, LA, and AA. HCl was used as one of the strong acids to weak acids (LA and AA). The alkaline medium was prepared at pH 8.0 by NO. The MVs were analyzed by SEM. Intact *S. mutans* with the MVs and bacterial forms broken by NO were observed, in contrast with the control group (Figure 1A). Various MVs particles, including small, aggregated, and forms, were observed in MVs from *S. mutans* grown under various conditions (Figure 1B). Small particles of MVs were observed in the HC, LA, and AA groups (Figure 1B,C). In contrast, MVs were larger in the NO group. To confirm whether these MVs were acquired from *S. mutans* grown under these conditions, the corresponding growth curves of *S. mutans* were evaluated. The growth of *S. mutans* was slower at an initial pH of 6.0 than in the control group, while at an initial pH of 8.0, the growth was similar to that of the control (Supplemental Figure S1A). Furthermore, cell growth under all initial pH 6.0 conditions similarly decreased to less than pH 5.0 in a time-dependent manner with culture (Supplemental Figure S1B). Under the initial pH 8.0 conditions, the pH decreased depending on the culture time, but the pH was always higher than that of the control, in which the culture was started at pH 7.2, and not decreased to pH 5.5.

A particle size rate of 0–4000 nm^2^ was mainly found in all MVs (Figure 1C). The MV particles of 200–500 nm^2^ were dominant in the control group (Control, pH 7.2), but a particle of 0–200 nm^2^ was predominant. In contrast, particle sizes of 0–200 nm^2^ were dominant in the MVs extracted from the initial conditions of pH 6.0 in the HC, LA, and AA groups, as well as in the NO group. A particle size of more than 1000 nm^2^ was observed in the NO group, which was higher than those in the other groups.

### 3.2. Biofilm Formation Induced by the Addition of MVs Samples from S. mutans Grown under Various Initial Conditions

To elucidate the effects of the initial pH on the biofilm induction abilities of the MVs produced from biofilm formation of *S. mutans*, UA159 *gtfBC^-^* was quantitatively assessed in TSB with 0.25% sucrose with various concentrations of MVs from *S. mutans* UA159 in initial pH 6.0 condition prepared with HC (A), LA (B), and AA (C), and the initial pH 8.0 condition prepared with NO (D), and compared with control (no preparation). The data indicate the mean ± standard deviation (SD) of three independent experiments. The asterisks indicate a significant difference between the two groups (*: *p* < 0.05, the MVs in the control condition vs the MVs in the initial pH 6.0 or 8.0 conditions).

**Figure 2:**
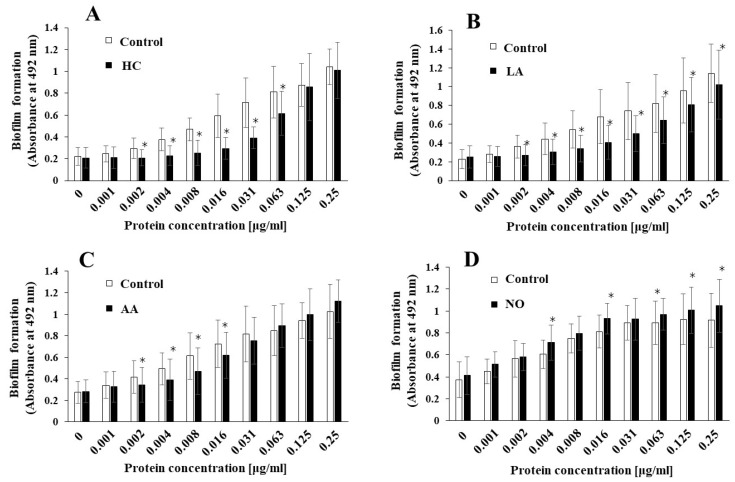
Effects of initial pH condition on the MVs-dependent biofilm formation. Biofilm formation of *S. mutans* UA159 *gtfBC^-^* was quantitatively assessed in TSB with 0.25% sucrose with various concentration of MVs from *S. mutans* UA159 in initial pH6.0 condition prepared with HC (**A**), LA (**B**) and AA (**C**), and initial pH 8.0 condition prepared with NO (**D**), and compared with control (no preparation). The data indicate the mean ± standard deviation (SD) of three independent experiments. The asterisks indicate a significant difference between the groups (*: *p* < 0.05, MVs in control condition vs. MVs in initial pH6.0 or 8.0 conditions).

*S. mutans* UA159, the MVs produced in the HC, LA, and AA, and NO groups were applied to cultures of *S. mutans gtfBC*^-^. Biofilm formation was induced by the addition of the MVs and was compared with that using control MVs in TSB with 0.25% sucrose. The MVs under the control conditions induced biofilm formation in a dose-dependent manner (Figure 2A–D). In contrast with that in the control group, the biofilm formation induced by the MVs under the initial pH 6.0 conditions was significantly lower in HC at 0.002–0.063 mg/mL (Figure 2A), LA at 0.002–0.25 mg/mL (Figure 2B), and AA at 0.002–0.016 mg/mL MVs (Figure 2C). The reduction in biofilm formation was reversed at high concentrations of the MVs prepared with HC and AA. However, the reduction in biofilm formation was continued at high concentrations of MVs prepared with LA. The biofilm formation induced by the MVs under the initial pH 8.0 conditions was significantly higher in the NO group, at 0.004, 0.016, and 0.063–0.25 mg/mL MVs (Figure 2D), than in the control group. These results suggested that the level of Gtfs might be decreased at the initial pH of 6.0, in contrast with the control MVs, because MV-dependent biofilm formation was induced by the presence of Gtfs in the MVs [11]. Conversely, the level of Gtfs might be increased by the initial pH 8.0 condition. Therefore, as a next step, the presence of Gtfs was confirmed by SDS-PAGE and CBB staining, and Gtf activity was also confirmed by zymography.

### 3.3. Role of Gtfs in Biofilm Formation Induced by the Addition of MVs

**Figure 3:**
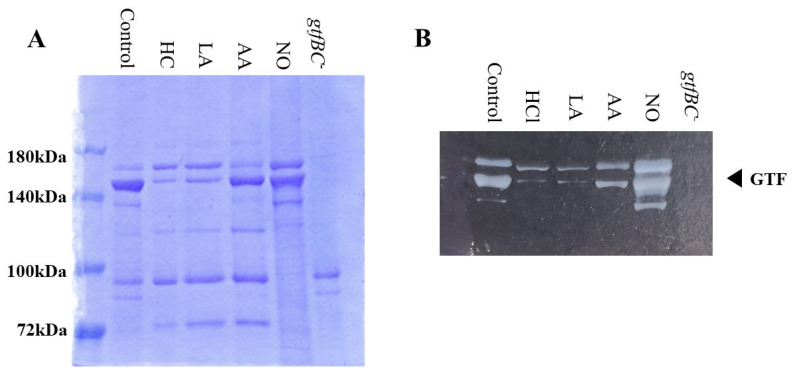
Effects of initial pH condition on Gtfs volume and activities in the MVs. Amounts of Gtfs on MVs extracted from *S. mutans* UA159 in control, HC, LA, AA, and NO were analyzed in 6% polyacrylamide gel, SDS-PAGE and CBB staining (**A**), and zymography using buffer involving sucrose (**B**). Representative data from more than three independent experiments are shown in the pictures.

**Figure 4:**
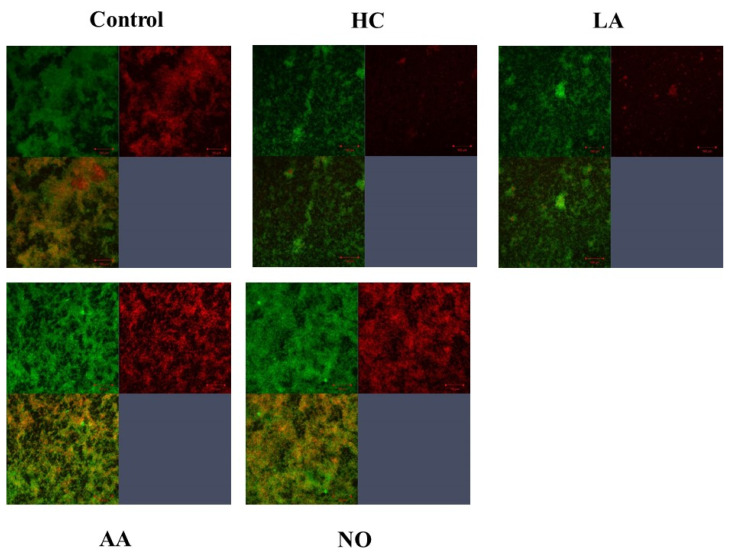
Confirmation of Gtfs on the MVs in the various conditions. To confirm glucan synthesis in biofilm formation induced by MVs that were extracted in control, HC, LA, AA and NO conditions, their biofilms were stained by SYTO 9 and Alexa Flour 647-dextran conjugate, observed by confocal microscope and analyzed by Zen. Upper left: live cells, upper right: glucan, and lower left: live cell merged with glucan were presented. Representative data from more than three independent experiments were presented in the pictures.

Gtf activities and a larger band at approximately 150 kDa were confirmed in control MVs by SDS-PAGE gel staining with CBB and zymography, respectively (Figure 3A,B). The Gtf bands were smaller in the MVs from the HC and LA groups than in those from the control group. Moreover, the Gtf bands were larger in the MVs from the initial pH 6.0 solutions prepared with AA than in the HC and LA groups, and smaller than those in the control MVs. In contrast, the Gtf bands were larger in the MVs from the NO group than those from the other groups. Therefore, the initial pH may control the expression of Gtfs on MVs. To confirm the expression of Gtfs in MVs, SDS-PAGE and Western blotting using anti-MV antibodies, which respond to Gtfs, were performed. It was confirmed that the protein band was Gtfs (Supplemental Figure S2). To confirm the effects of the initial pH conditions on the Gtf activities in the biofilm formation, glucan was stained with Alexa Fluor 647-dextran conjugate and observed under confocal microscopy in biofilms induced with the MVs under conditions with 0.25% sucrose. The MVs from the HC and LA groups could not induce clear glucan formation, but the MVs from the AA and NO groups presented glucan formation as well as the control group (Figure 4).

**Figure 5:**
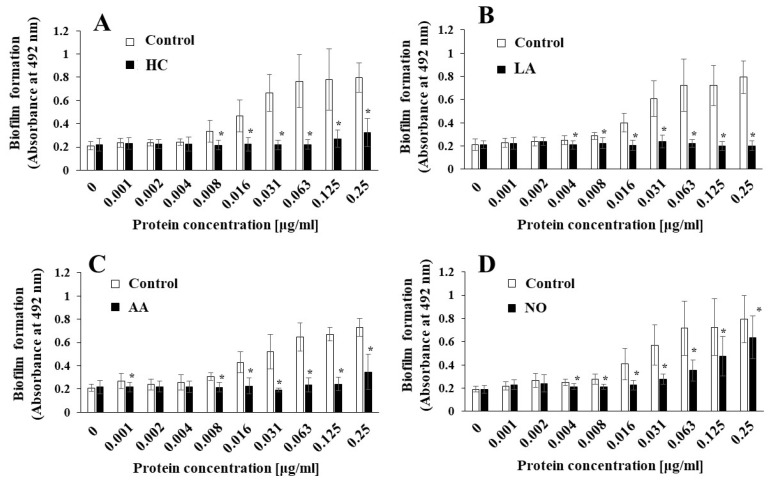
Role of Gtfs for effects of initial pH condition on the MVs-dependent biofilm formation. Biofilm formation of *S. mutans* UA159 *gtfBC^-^* was quantitatively assessed in TSB with 0.25% sucrose with various concentration of MVs from *S. mutans SMU833^-^* in initial pH 6.0 condition prepared with HCl (**A**), LA (**B**), and AA (**C**), and initial pH 8.0 condition prepared with NO (**D**). The data indicate the mean ± SD of three independent experiments. The asterisks indicate a significant difference between the groups (*: *p* < 0.05, MVs in control condition vs MVs in initial pH 6.0 and 8.0 conditions).

Oral biofilms consist of various oral bacteria, generating low pH conditions in the presence of sugars. To study whether the change in the initial pH affects the MV-dependent biofilm formation of other oral bacteria, these MVs were applied to cultures of *S. mitis* ATCC 903 and *A. naeslundii* x600, which are the initial colonizers on the tooth surface. The MV-dependent biofilm formation levels of *S. mitis* were significantly inhibited by HC at 0.125 and 0.25 μg/mL, and by LA at 0.008–0.25 μg/mL compared with the control (Supplemental Figure S3A,B). However, AA did not affect biofilm formation (Supplemental Figure S3C). In contrast, the biofilm formation levels were significantly increased at high concentrations (0.063–0.25 μg/mL) of the MVs prepared in the presence of NO (Supplemental Figure S3D). The biofilm formation levels of *A. naeslundii* were significantly inhibited by the HC and LA groups at high concentrations (0.063–0.5 μg/mL) (Supplemental Figure S4A,B). There were no significant differences at any concentration in the AA and NO groups (Supplemental Figure S4C,D). Taken together, MV-dependent biofilm formation was reduced by HC and LA. The degree of Gtf expression on the MVs was considered a reason for the reduction in biofilms. However, comparing the HC, LA, and AA groups under the same pH 6.0 conditions, the AA group induced different phenotypes of Gtf expression and different activities in the biofilm of oral bacteria. To clarify the role of Gtfs in MV-dependent biofilm formation, *S. mutans* SMU833^-^, whose Gtf levels are naturally and systemically decreased [44], was used to provide MVs with lowered Gtf activities for the biofilm formation of *S. mutans* UA159.*gtfBC^-^*. The MVs did not stimulate the biofilm formation of UA159.*gtfBC*^-^ in the HC, LA, and AA groups, but stimulated biofilm formation in the NO group at higher concentrations (Figure 5). The biofilm formation levels were significantly lower in MVs from the NO group than in those from the control (Figure 5D).

**Figure 6:**
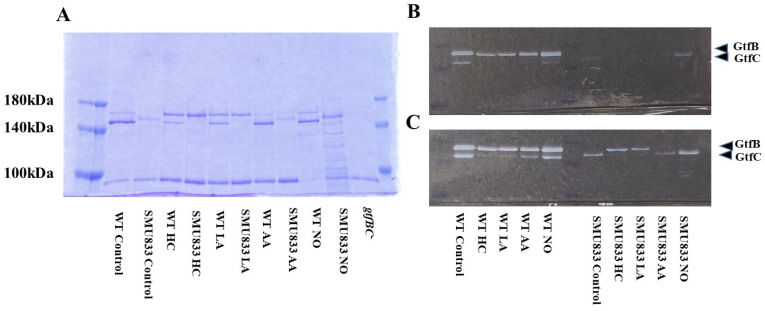
Effects of initial pH condition on Gtfs amounts and activities in the MVs from *S. mutans SMU833^-.^.* Amounts of Gtfs (GtfB and GtfC) on MVs from *S. mutans* UA159 (WT) and *S. mutans* UA159.*SMU833^-^* extracted in control, HC, LA, AA, and NO, and *S. mutans* UA159 *gtfBC^-^* were analyzed in 6% polyacrylamide gel, SDS-PAGE and CBB staining (**A**), and zymography for overnight (**B**) and three days culture (**C**) using buffer involving sucrose. Representative data from more than three independent experiments are shown in the pictures.

To observe Gtf activities in MVs of *S. mutans SMU833^-^* prepared from initial pH 6.0 and pH 8.0 conditions, SDS-PAGE and zymography were performed. The GtfC band at approximately 150 kDa was slightly expressed and shifted toward higher molecular weights in the MVs from *S. mutans SMU833^-^* than in those from the wildtype *S. mutans* under control conditions (Figure 6A). The GtfC band disappeared in the HC and LA groups, but more molecules were strongly expressed in the 165 kDa band of GtfB than of GtfC. The GtfC activities in the HC and LA groups were extremely reduced at 16 h and 3 days, and GtfB activities were observed in *S. mutans SMU833^-^* compared with the control at 3 days of culture by zymography (Figure 6B,C). A slightly shifted GtfC band and Gtf activity were found at 3 days of culture in the AA group (Figure 6C). The GtfC band and Gtf activity were strongly present at three days of culture in the NO group. The Gtf activity slightly induced biofilm formation at high concentrations of MVs from the NO group (Figure 5D). In contrast, the initial pH 6.0 conditions inhibited the expression and activity of GtfC. Therefore, the amounts of Gtf contained in the MVs from *S. mutans SMU833^-^* were systemically lost at an initial pH of 6.0, and inhibited the biofilm formation of *S. mutans* UA159.*gtfBC*^-^.

**Figure 7:**
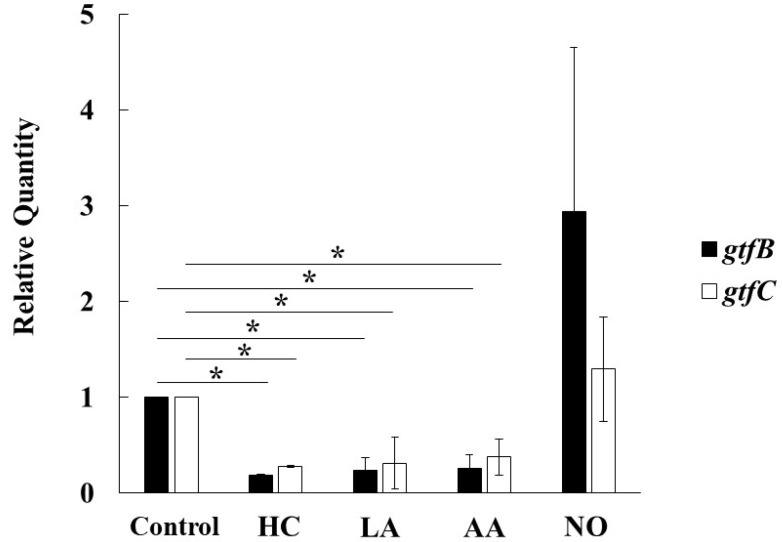
Effects of initial pH condition on expression of *gtfB* and *gtfC* in the MVs. Expression of *gtfB* and *gtfC* was quantitatively assessed by real-time PCR in 6 h incubation of *S. mutans* UA159 in initial pH 6.0 condition prepared with HC, LA and AA, and initial pH 8.0 condition prepared with NO, and compared with control condition. The data of relative expression in test conditions to control condition indicate the mean ± SD of three independent experiments. The asterisks indicate a significant difference between the groups (*: *p* < 0.05, MVs in control condition vs MVs in initial pH 6.0 and 8.0 conditions).

To observe the genetic expression of *gtf* genes, real-time PCR was performed in *S. mutans* UA159 cultivated for 6 h under various culture conditions. The expression of *gtfB* and *gtfC* was inhibited by HC, LA, and AA in *S. mutans* (Figure 7). In contrast, the expression of *gtfB* and *gtfC* was extremely and slightly increased, respectively, in the NO group compared with the control. Therefore, the Gtf expression is key for MV-dependent biofilm formation controlled by the initial pH conditions.

### 3.4. Search for Other Factors in Biofilm Development Induced by the Addition of MVs

**Figure 8:**
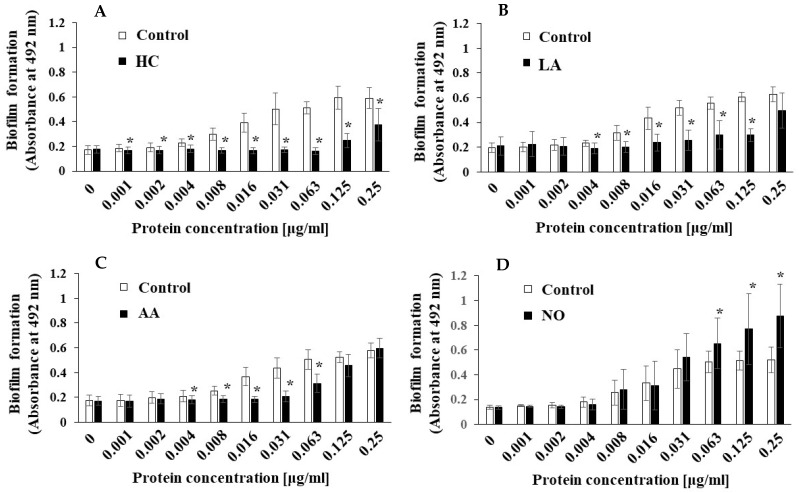
Effects of initial pH condition on the MVs-dependent biofilm formation in condition with raffinose. Biofilm formation of *S. mutans* UA159 *gtfBC^-^* was quantitatively assessed in TSB with 0.25% raffinose with various concentrations of MVs from *S. mutans* UA159 in initial pH 6.0 condition prepared with HC (**A**), LA (**B**), and AA (**C**), and the initial pH 8.0 condition prepared with NO (**D**). The data indicate the mean ± SD of three independent experiments. The asterisks indicate a significant difference between the groups (*: *p* < 0.05, MVs in control condition vs. MVs in initial pH 6.0 and 8.0 conditions).

To clarify the difference between the mechanisms of biofilm development by MVs under the initial pH 6.0 and pH 8.0 conditions, we searched for factors other than Gtfs. In our previous report [43], we found that raffinose induced a Gtf-independent and extracellular DNA (eDNA)-dependent biofilm formation of *S. mutans* UA159. The biofilm formation of UA159*.gtfBC*^-^ using MVs from *S. mutans* UA159 under various culture conditions was performed in TSB with 0.25% raffinose. The MV-dependent biofilm formation levels were significantly inhibited by HC and LA at various concentrations of MVs compared with those under the control conditions (Figure 8AB). The biofilm formation levels were significantly inhibited by AA at intermediate concentrations (0.004–0.063 μg/mL), but the inhibition returned to the control level at higher concentrations (0.125 and 0.25 μg/mL). Compared with those in the control group, the biofilm formation levels in the NO group were significantly upregulated by the MVs at higher concentrations (0.063–0.25 μg/mL). These results were similar in both TSB with 0.25% raffinose and TSB with 0.25% sucrose. To confirm the dependencies of Gtfs on biofilm formation, glucan formed in biofilms was labeled with an Alexa Fluor 647-dextran conjugate and observed by confocal microscopy. However, glucan formation was not observed in the biofilm formed by MVs extracted from the control, AA, and NO groups in TSB with raffinose (Supplemental Figure S5).

In TSB with 0.25% raffinose, fructan was synthesized by fructosyltransferase (Ftf) in the biofilm of *S. mutans* [43]. To search for the presence of Ftf (levansucrase) in these MVs, TOF-MS analyses were performed on two dominant protein bands of approximately 90 kDa and 45 kDa (not from Gtfs) in SDS-PAGE using the MVs extracted from conditions prepared with LA and NO (Supplemental Figure S6). From the results, the band at approximately 90 kDa was presumed to be a levansucrase ftf gene. This band was strongly expressed in the HC, LA, and AA groups compared with the control group. It was also confirmed that these proteins had Ftf activities by zymography overnight, or after three days of incubation (Supplemental Figure S7). Levansucrase produces fructan and may be associated with the development of biofilms [43]. However, the protein expression levels of Ftf were not correlated with biofilm formation in these MVs (Figure 8 and Supplemental Figure S7). The MVs from *S. mutans* UA159.*sacB*^-^, in which Ftf activities were lacking, did not show bands or activities at approximately 90 kDa. Interestingly, bands at approximately 150 Da were slightly observed, which might be GtfC, in the control, AA, and NO groups after seven days of culture, but not overnight (Supplemental Figure S7C). Gtf activities were slightly found in the control, AA, and NO groups. The band at approximately 45 kDa was presumed to be an elongation factor Tu and tuf gene in only the NO group (Supplemental Figure S6). However, this protein was not considered to be systemically involved in biofilm formation.

**Figure 9:**
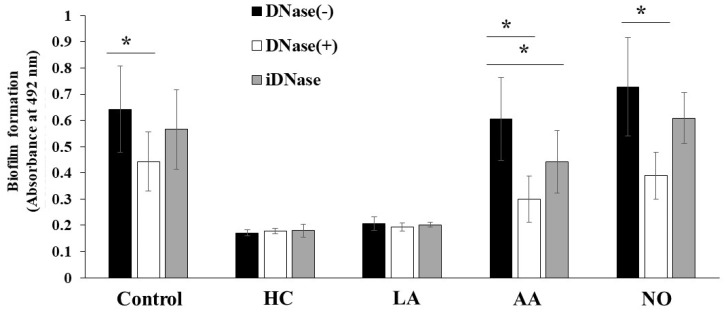
Role of eDNA for effects of initial pH condition on the MVs-dependent biofilm formation. Biofilm formation of *S. mutans* UA159 *gtfBC*^-^ was quantitatively assessed in TSB with 0.25% raffinose with 0.125 mg/ ml of MVs from *S. mutans* UA159 in HC, LA, AA and NO, with or without DNase I and iDNase I. The data indicate the mean ± SD of three independent experiments. The asterisks indicate a significant difference between the groups (*: *p* < 0.05, MVs in condition without DNase-I vs MVs in condition with DNase-I or iDNase-I).

The biofilm formation of *S. mutans* UA159 was eDNA-dependent in TSB with 0.25% raffinose [43]. To confirm whether these biofilm formations are eDNA-dependent, DNase I was added to the cultures, and biofilm formation was observed after 6 h of culture. The MV-dependent biofilm formation was half-inhibited by DNase I in the AA and NO groups (Figure 9). In contrast, the MV-dependent biofilm formation was inhibited by 1/3 with DNase I under the control conditions. The inhibition by DNase I was returned to the control level by heating to DNase I. Therefore, the main mechanism of MV-dependent biofilm formation was eDNA dependence in TSB with 0.25% raffinose in the AA and NO groups. There were two mechanisms using Gtf and eDNA for the biofilm formation induced with MVs from *S. mutans* grown in condition with the initial pH.

## 4. Discussion

The study of gram-positive bacteria-derived vesicles is rapidly expanding and suggests a newfound pathogenicity of oral bacterial infection. Recently, various reports disclosed that MVs from gram-positive bacteria enclosed many virulence-related proteins such as streptolysin O and NAD-glycohydrolase in *Streptococcus pyogenesis* [45] and lipoproteins in *Staphylococcus aureus* [46] and *Streptococcus pneumoniae* [47]. MVs play roles in intercellular communication involving biofilm formation, nutrition acquisition and quorum sensing [9,47,48,49].

The cargo of the vesicle reflects the physical state of the cell at the time of death rather than being the consequence of a specific sorting mechanism [50]. Rainey et al. indicated that changes in gene expression under acidic pH conditions might lead to activated secretion of MVs and eDNA [51]. In our previous report [37], *S. mutans* formed significant biofilms after 16 h of culture under primary culture at pH 6.0, which showed a lower production of insoluble glucan than that under primary culture at pH 7.0. The Gtf activities were reduced after 4 h of culture in the primary pH 6.0 condition compared with the primary pH 7.0 condition. Therefore, pH conditions may be a key factor in producing various characteristics of MVs from *S. mutans* and control Gtfs. In a more recent paper, proteomic and metabolomics analyses of the MVs from *S. mutans* UA159 at initial pH values of 7.5 and 5.5 were performed [31]. Alterations in proteins involving ABC transporters were recognized in MVs under acid pressure. A large number of transporter are apparently involved in the uptake of carbohydrate associated with cariogenesis in *S. mutans*. In contrast, the initial pH 8.0 conditions prepared with NaOH enhanced the expression of *gtfB* and *gtfC* and the protein levels of GtfB and GtfC in the MVs compared with those in the control. Under the initial pH 8.0 conditions, the pH did not decrease to less than pH 5.5 during 16 h of culture, and there is a possibility that the expression of *gtfB* and *gtfC* was not inhibited by low pH. We confirmed a higher expression of *gtfB* and *gtfC* at 6 h after culture in the NO group than in the HC, LA, and AA groups. Therefore, it was considered that larger amounts of Gtfs were loaded on the MVs in the NO group. The size of the MVs was also increased by the presence of Gtfs because Gtfs are likely adhesion factors for MV-to-MV bonding [19]. The quantitative change in insoluble and soluble glucans between primary pH 6.0 and pH 8.0 associated with different activities on MV-dependent biofilm formation resulted in a change in biofilm quantity.

A previous report showed that *Bacillus subtilis* death results from a change in the intracellular pH caused by the passage of weak organic acids, such as acetic acid, across the cell membrane when incubated with *B. subtilis* at a pH value near the pKa (4.7) [52]. These starved plaque fluid samples predominantly contained lactic acid, butyric acid, propionic acid, and acetic acid with high pKa (dissociation constant) values of 3.86, 4.82, 4.87, and 4.76, respectively [53]. In the culture of *S. mutans* in the BHI broth, the pH decreased to approximately 4.5 under the initial pH of 6.0. According to the Stephan curve, the pH in dental plaques decreases to less than 5.0 after every meal [25] and never to less than pH 4.0. Weak acids, such as acetic acid, can permeate bacterial membranes more readily than strong acids, such as HCl, and lactic acid, with pKa values of −3.7 and 3.86, due to the equilibrium between their ionized and nonionized forms at the pKa [54]. The expression of the *gtfB* and *gtfC* genes was reduced at 6 h after culture under the initial pH 6.0 conditions prepared with all acids, but in the AA group, the protein expression of GtfC was higher than that in the HC and LA groups. These nonionized forms pass through the bacterial membrane, and protons induce acid tolerance responses in cells. Proton-pumping F_1_-F_0_ ATPase is essential for the maintenance of intracellular pH conditions that are favorable for *S. mutans* [34,35]. Other responses include the production of ammonia via the agmatine deiminase system [55], alterations in metabolic pathways [56], and low-pH-dependent changes in membrane fatty acid composition [57]. Gtfs might be slightly loaded on MVs because proton stress by acetic acid might induce acid tolerance responses such as an increase in proton-pumping F_1_-F_0_ ATPase, providing MVs. The role of acetic acid may be different from the role of lactic acid in the development of MV-dependent biofilm formation because lactic acid cannot undergo conversion to its nonionized form from its ionized form at pH 4.5 under culture conditions and in dental plaques. The ionized form affects the bacterial membrane surface and might inhibit the loading of *gtfB* and *gtfC* on MVs under low-pH-dependent changes in membrane fatty acid composition. However, the difference in GtfC expression on MVs between preparations with lactic acid and acetic acid has not been fully elucidated; further study of the relationship between acid tolerance responses and the expression mechanisms of proteins in the MVs prepared with ionized and nonionized acid forms is necessary to completely understand MV-dependent biofilm formation.

Rhamnose-glucose polysaccharide (RGP) is a component of the cell wall in *S. mutans* and is linked to peptidoglycan. RGP is important for fitness under stress conditions and the maintenance of virulence traits, and is also involved in antibiotic resistance [58,59]. Glucose side chains are formed by Gtf, RgpE, RgpH, and RgpI. The deletion of *rgpI* (SMU833) was previously shown to decrease the amounts of Gtfs and increase eDNA levels [44]. We confirmed that the Δ*rgpH* and Δ*rgpI* (*SMU833* mutant) strains could form eDNA-dependent biofilms [40]. However, the biofilm formation levels were slightly induced in the NO groups, but not significantly induced in the HC, LA, and AA groups. Therefore, it was suggested that the decreased levels of Gtfs strongly guided low levels of MV-dependent biofilm formation and negated the positive effects of the initial pH on biofilm formation in the *S. mutans SMU833* mutant. The MVs from the *S. mutans SMU833* mutant slightly induced eDNA-dependent biofilm formation by a small amount of GtfC in the NO groups.

Slight sucrose contamination has been found in TSB [41]. Collective behaviors, such as biofilm formation and quorum sensing, are affected by carbohydrate sources such as sucrose [60,61]. Cell attachment and aggregation in TSB with 0.25% raffinose are induced by a mechanism whereby the cell surface hydrophobicity is increased by the activity of GtfC using sucrose contamination in TSB [41]. In zymography, the activities of Gtfs on these MVs from *S. mutans* were not confirmed overnight, but were confirmed at three days after incubation in TSB with 0.25% raffinose. Therefore, it was considered that a slight concentration of sucrose induced the Gtf activities to produce glucan. Even if sugar was present in the medium, a slight concentration of sucrose did not induce Gtf-dependent biofilm formation because the volume was insufficient to form a biofilm. In contrast, eDNA-dependent biofilms might be increased by the slight presence of Gtfs. Previously, we reported that eDNA detected in the biofilm formed in TSB with 0.25% raffinose was likely genomic DNA released from dead cells. This result agreed with other reports showing that eDNA generated by cell lysis was important for biofilm formation by *S. mutans* [62]. Therefore, a small amount of Gtfs may increase the hydrophobicity, destroy cells, and expose DNA. eDNA is released by multiple mechanisms such as the lysis of subpopulations of bacterial cells mediated by prophages, lytic proteins, and enzymes, [63,64] and via active release by bacterial MVs [10,65,66]. These effects trigger the initial stage of biofilm formation and determine the biofilm architecture [64,67]. The MVs induce the cell attachment and aggregation of multiple species by larger amounts of Gtfs and induce eDNA to promote the primary attachment of initial colonizers on tooth surfaces by small amounts of Gtfs [11,43].

In our previous report, the participation of fructan was associated with cell aggregates in *S. mutans* UA159. Levan, which is a type of fructan, was reported to enhance the viscosity of DNA solutions, and was necessary for biofilm formation by *Bacillus subtilis* [68]. However, the presence of Ftf in the MVs from *S. mutans* was not associated with MV-dependent biofilm formation in TSB with 0.25% raffinose. Therefore, the presence of Ftf in the MVs did not affect MV-dependent biofilm formation. Disruption of the *ftf* gene leads to a decrease in virulence to 70% of the cariogenic activity of the wild type strain, which is measured as the ability to produce caries in gnotobiotic rats [69]. Reduced fructan accumulation in the mutant strain may affect virulence in *S. mutans* because fructans are commonly used by this species as a reserve carbohydrate. Therefore, fructan may indirectly contribute to MV-dependent biofilm formation.

## 5. Conclusions

The initial pH conditions affect the characteristics of the MVs from *S. mutans* to formation biofilms due to the altered expression of Gtfs. This finding suggests that environmental conditions determine the differences in pathogenic activities locally during biofilm development in the oral cavity. These findings help to increase the scientific perspective in our knowledge of *S. mutans* MVs, where the MVs may play an important role in dental caries. It may be of importance to develop biofilm removers targeting the MVs in the next generation for oral hygiene. At that time, a procedure of controlling the pH will be necessary for the development of effective biofilm removers.

## Figures and Tables

**Table 1:** Primers for qPCR.

Primers	Sequences
Ldh-Fw	TTGGCGACGCTCTTGATCTTAG
Ldh-Rv	GTCAGCATCCGCACAGTCTTC
GTF-BF	CGAAATCCCAAATTTCTAATGA
GTF-BR	TGTTTCCCCAACAGTATAAGGA
GTF-CF	ACCAACCGCCACTGTTACT
GTF-CR	AACGGTTTACCGCTTTTGAT

## Data Availability

Not applicable.

## Supplementary Materials

The following are available online at https://www.mdpi.com/article/10.3390/microorganisms9112308/s1. Figure S1: Effects of initial pH condition on the growth of *S. mutans* and change of pH during the culture, Figure S2: Effects of initial pH condition on expression of GtfB and GtfC in MVs, Figure S3: Effects of initial pH condition on the MVs-dependent biofilm formation of *S. mitis*. Figure S4: Effects of initial pH condition on the Mvs-dependent biofilm formation of *A. naeslundii*, Figure S5: Confirmation of glucan in the biofilm formation induced with MVs, Figure S6: Effects of initial pH condition on expression of GtfB and GtfC in MVs, Figure S7: Effects of initial pH condition on Ftf volume and activities in the MVs from *S. mutans.*
