# Supplementary material for: Effects of pH on the Properties of Membrane Vesicles Including Glucosyltransferase in Streptococcus mutans
Source: Microorganisms. 2021 Nov 6;9(11):2308. doi: 10.3390/microorganisms9112308 (PMC8618110; doi:10.3390/microorganisms9112308)
Supplement: Supplementary file 1 [file microorganisms-09-02308-s001.zip › microorganisms-1402398-supplementary.pdf]

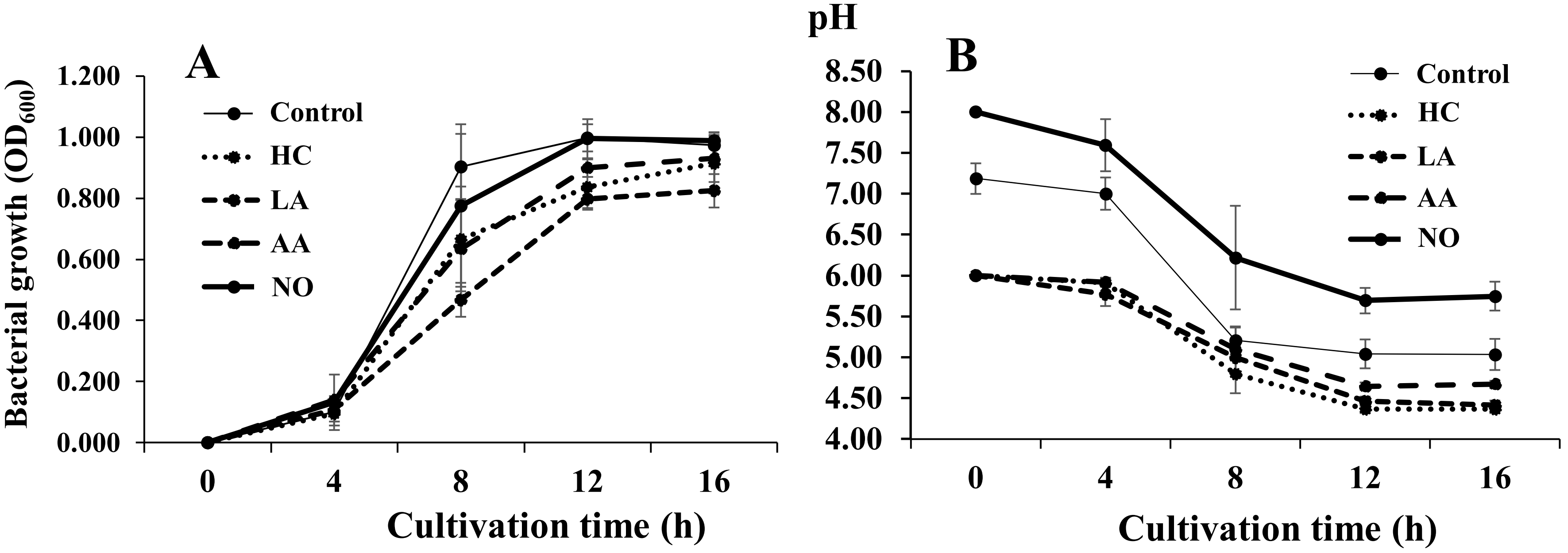

A
Bacterial growth (OD_{600})
1.200
1.000
0.800
0.600
0.400
0.200
0.000
0
4
8
12
16
Cultivation time (h)
Control
HC
LA
AA
NO
pH
B
8.50
8.00
7.50
7.00
6.50
6.00
5.50
5.00
4.50
4.00
0
4
8
12
16
Cultivation time (h)
Control
HC
LA
AA
NO

Figure S1. Effects of initial pH condition on the growth of S. *mutans* and change of pH during the culture

Growth of *S. mutans* UA159 and pH was quantitatively assessed in initial pH6.0 BHI condition prepared with control (no acid), HCl (HC), lactic acid (LA) and acetic acid (AA), and initial pH8.0 BHI condition prepared with NaOH (NO). The data indicate the mean ± standard deviation (SD) of three independent experiments.

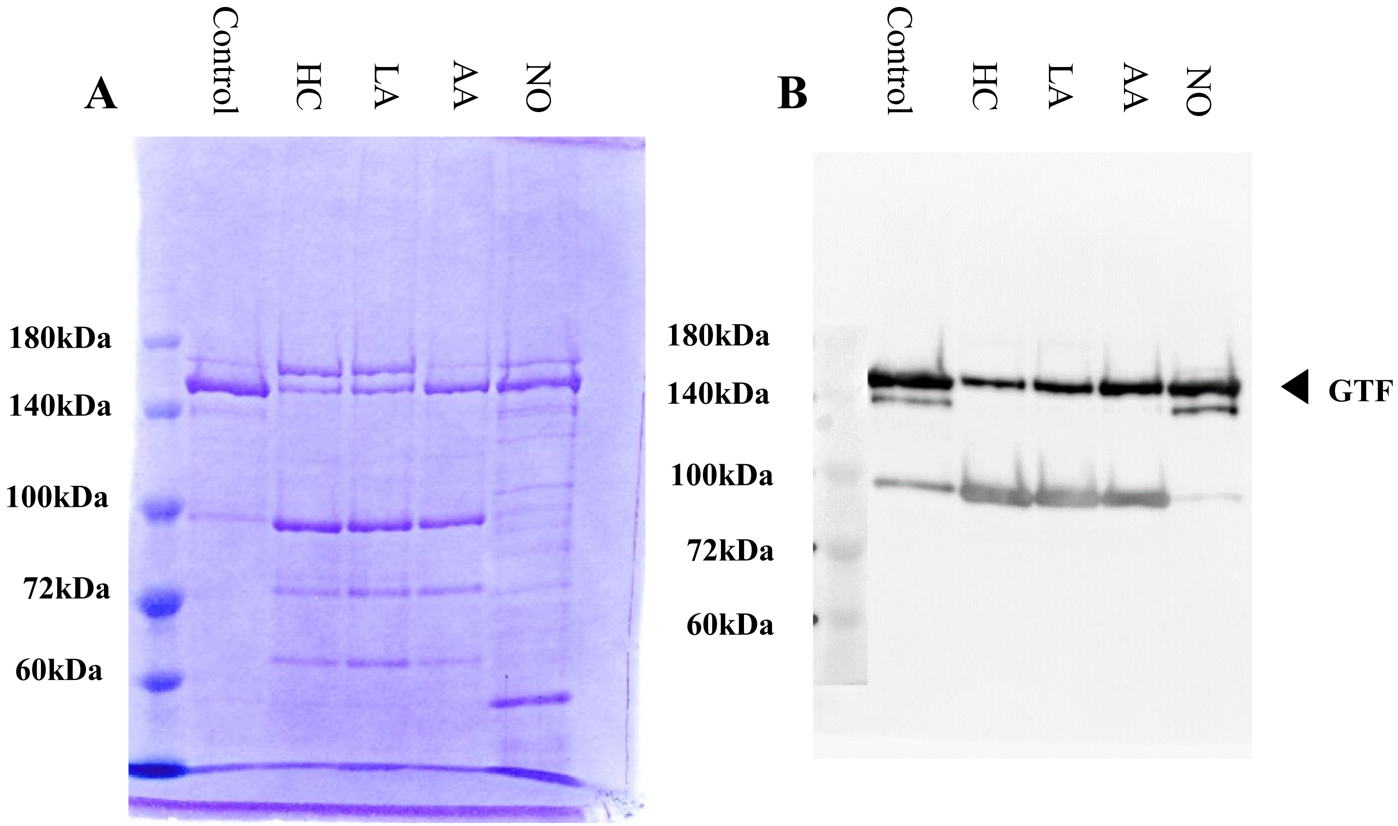
A
Control
HC
LA
AA
NO
180kDa
140kDa
100kDa
72kDa
60kDa
B
Control
HC
LA
AA
NO
180kDa
140kDa
100kDa
72kDa
60kDa
GTF

Figure S2. Effects of initial pH condition on expression of GtfB and GtfC in MVs.

Expression of GtfB and GtfC in MVs was quantitatively assessed in initial pH6.0 BHI condition prepared with control (no acid), HC, LA, AA, and NO. MVs from *S. mutans* UA159 was subjected to 6% polyacrylamide gel, SDS-PAGE and CBB staining (A). Furthermore, western blotting using anti-GTF antibodies was performed (B). Arrow indicated molecular size of Gtfs. Representative data from three independent experiments are shown in the pictures.

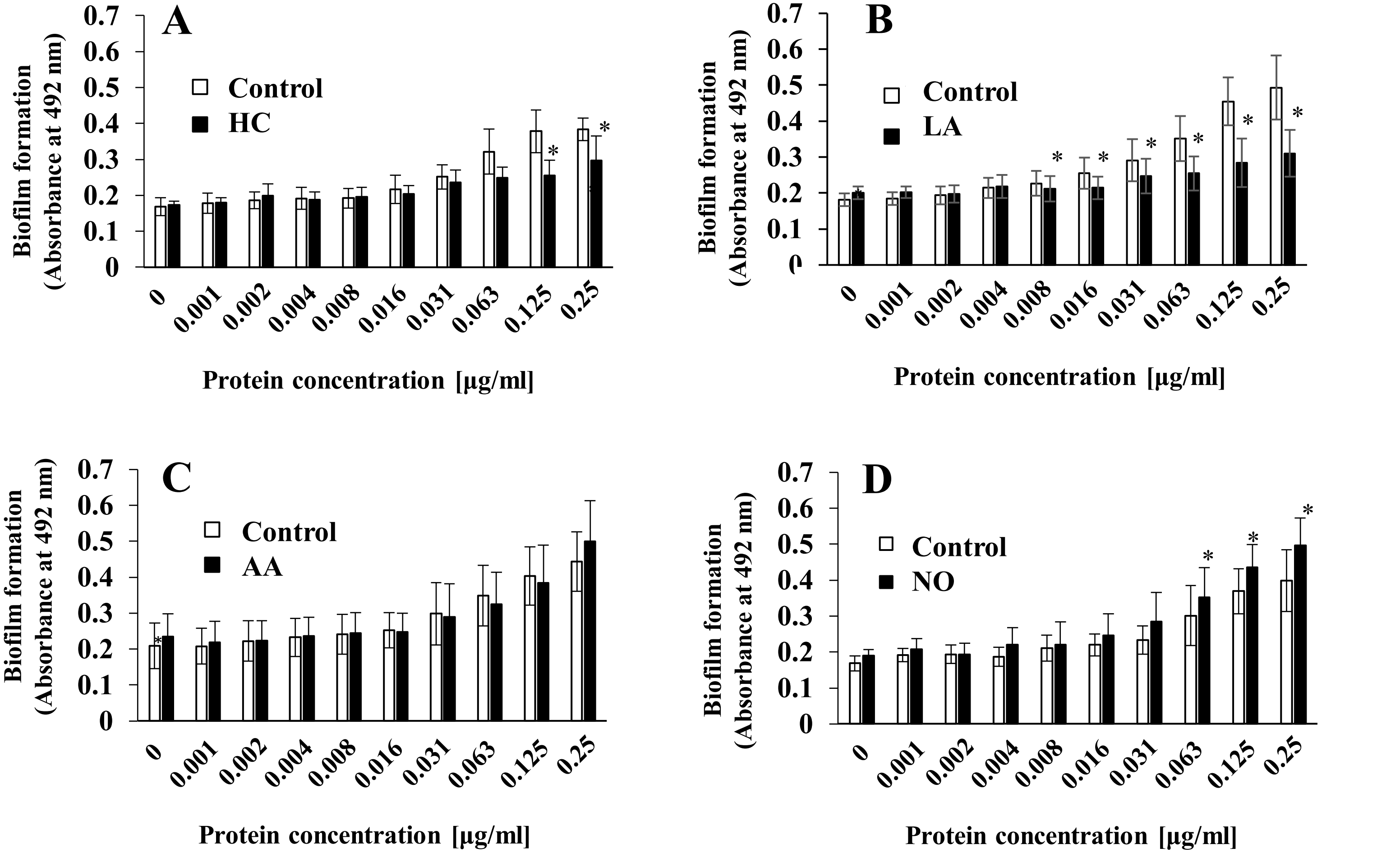
A
B
C
D
Control
HC
LA
AA
NO
Biofilm formation (Absorbance at 492 nm)
0
0.1
0.2
0.3
0.4
0.5
0.6
0.7
0
0.001
0.002
0.004
0.008
0.016
0.031
0.063
0.125
0.25
Protein concentration [µg/ml]

Figure S3. Effects of initial pH condition on the MVs-dependent biofilm formation of *S. mitis*

Biofilm formation of *S. mitis* ATCC 903 was quantitatively assessed in TSB with 0.25% sucrose with various concentration of MVs from *S. mutans* UA159 in HC (A), LA (B), AA (C), and NO (NO), and compared with MVs in control (no preparation). The data indicate the mean ± standard deviation (SD) of three independent experiments. The asterisks indicate a significant difference between the two groups (*: $p < 0.05$, MVs in control condition vs MVs in initial pH6.0 or 8.0 conditions).

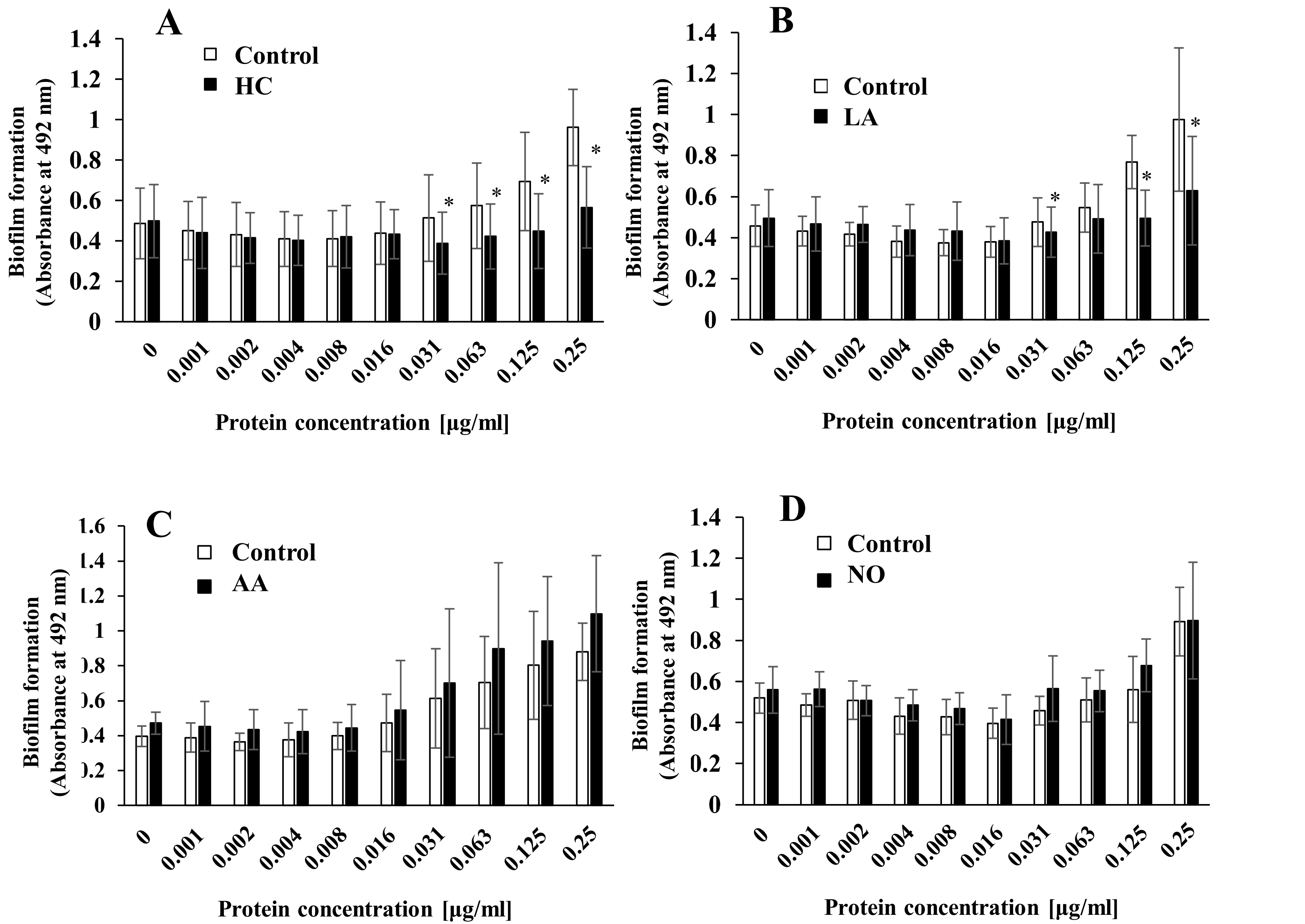

A
Control
HC
B
Control
LA
C
Control
AA
D
Control
NO
Biofilm formation
(Absorbance at 492 nm)
Protein concentration [µg/ml]
0
0.001
0.002
0.004
0.008
0.016
0.031
0.063
0.125
0.25

Figure S4. Effects of initial pH condition on the MVs-dependent biofilm formation of *A. naeslundii*

Biofilm formation of *A. naeslundii* x600 was quantitatively assessed in TSB with 0.25% sucrose with various concentration of MVs from *S. mutans* UA159 in HC (A), LA (B), AA (C), and NO (NO), and compared with MVs in control. The data indicate the mean ± standard deviation (SD) of three independent experiments. The asterisks indicate a significant difference between the two groups (*: $p < 0.05$, MVs in control condition vs MVs in initial pH6.0 or 8.0 conditions).

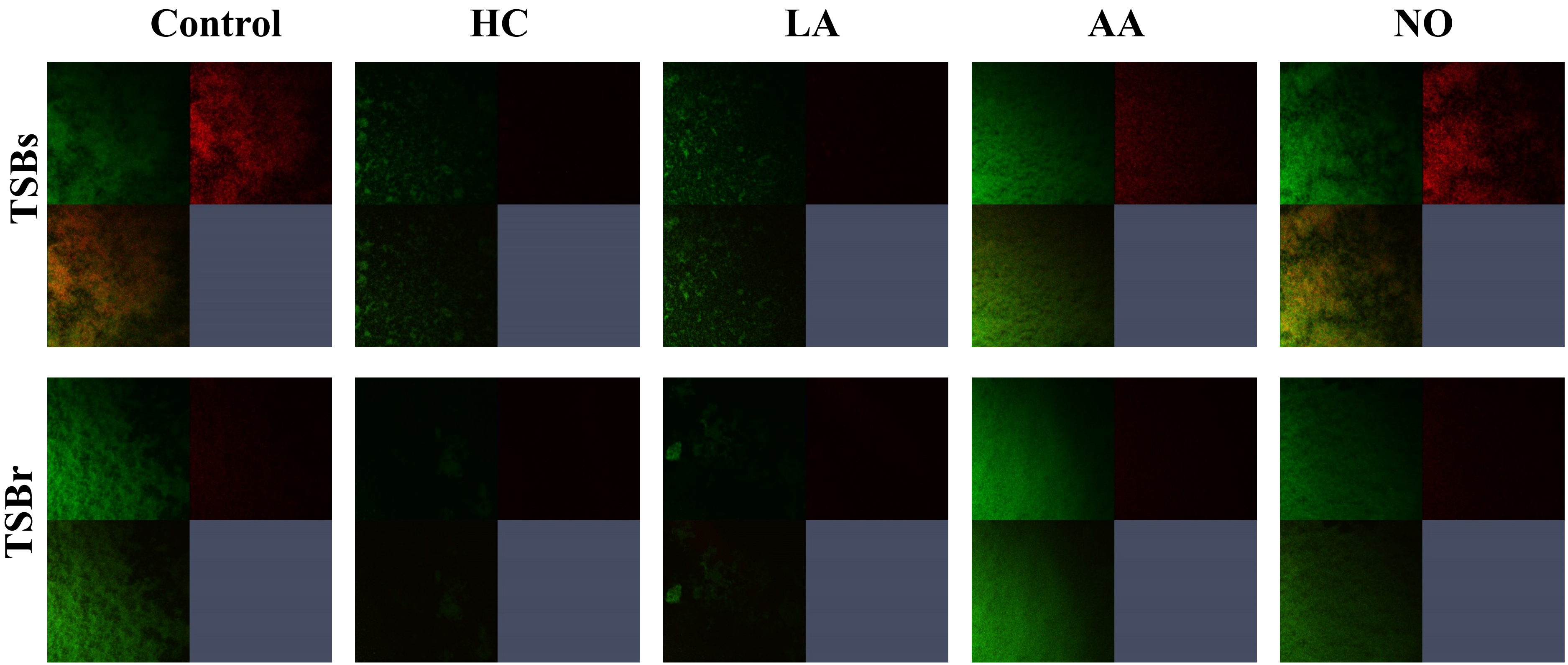
Control
HC
LA
AA
NO
TSBs
TSBr

Figure S5. Confirmation of glucan in the biofilm formation induced with MVs.

MVs in control condition, HC, LA, AA, and NO were applied to the biofilm formation assay using *S. mutans* UA159.*gtfBC*$^{-}$. To confirm the dependencies of Gtfs on biofilm formation, glucan formed in biofilms was labeled with Alexa Fluor 647-dextran conjugate and observed by confocal microscopy. After SYTO 9 staining to live cells, biofilms were observed by confocal microscopy. The upper left of the image shows live cells, the upper right shows glucan formation, and the lower left shows live cells and glucan formation. Representative data from more than three independent experiments are presented in the pictures.

TOF-MAS(PMF)
2020.10.21

6% polyacrylamide gel
MV SDS-PAGE

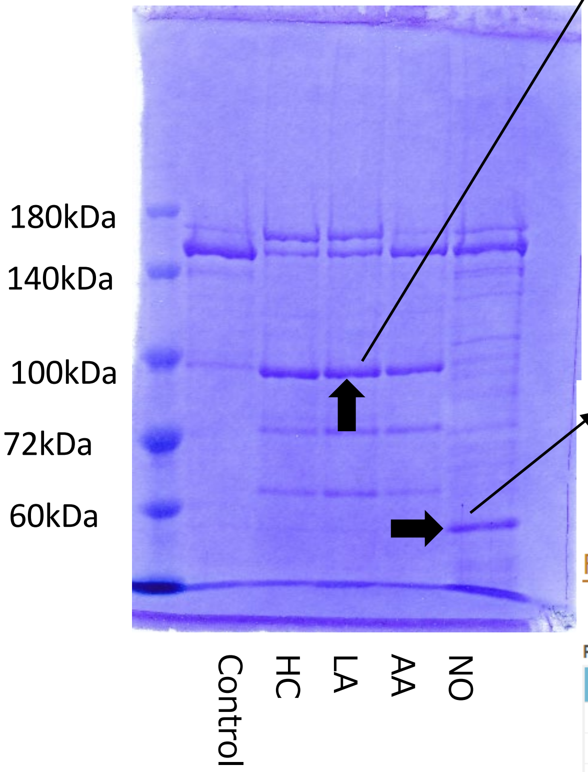

Length:795
Mass (Da):87,385
(Secreted)

Protein **Levansucrase**
Gene **ftf**
Organism *Streptococcus mutans serotype c (strain ATCC 700610 / UA159)*
Status Reviewed - Annotation score: ●●●○○ - Protein inferred from homology

Function

Catalytic activity

- [6)-β-D-fructofuranosyl-(2→]$_{(n)}$ α-D-glucopyranoside + sucrose = [6)-β-D-fructofuranosyl-(2→]$_{(n+1)}$ α-D-glucopyranoside + D-glucose
  EC:2.4.1.10
  Source: Rhea. « Hide

Length:398
Mass (Da):43,919
(Cytoplasm)

Protein **Elongation factor Tu**
Gene **tuf**
Organism *Streptococcus mutans serotype c (strain ATCC 700610 / UA159)*
Status Reviewed - Annotation score: ●●●○○ - Protein inferred from homology

Function

This protein promotes the GTP-dependent binding of aminoacyl-tRNA to the A-site of ribosomes during protein biosynthesis. UniRule annotation

Regions

Feature key	Position(s)	Description	Actions	Graphical view	Length
Nucleotide binding	19 – 26	GTP UniRule annotation			8
Nucleotide binding	84 – 88	GTP UniRule annotation			5
Nucleotide binding	139 – 142	GTP UniRule annotation			4

Figure S6. Effects of initial pH condition on expression of GtfB and GtfC in MVs.

MVs in control, and HC, LA, AA, and NO conditions were applied to the biofilm formation assay using 6% polyacrylamide gel, SDS-PAGE and CBB staining, and prominent protein bands (at approximately 90 kDa and 50 kDa) were analyzed by PMF analysis using matrix-assisted laser desorption/ionization time-of-flight (MALDI-TOF) mass spectrometry. Levanucrase and elongation factor Tu were estimated at 90 kDa and 50 kDa, respectively.

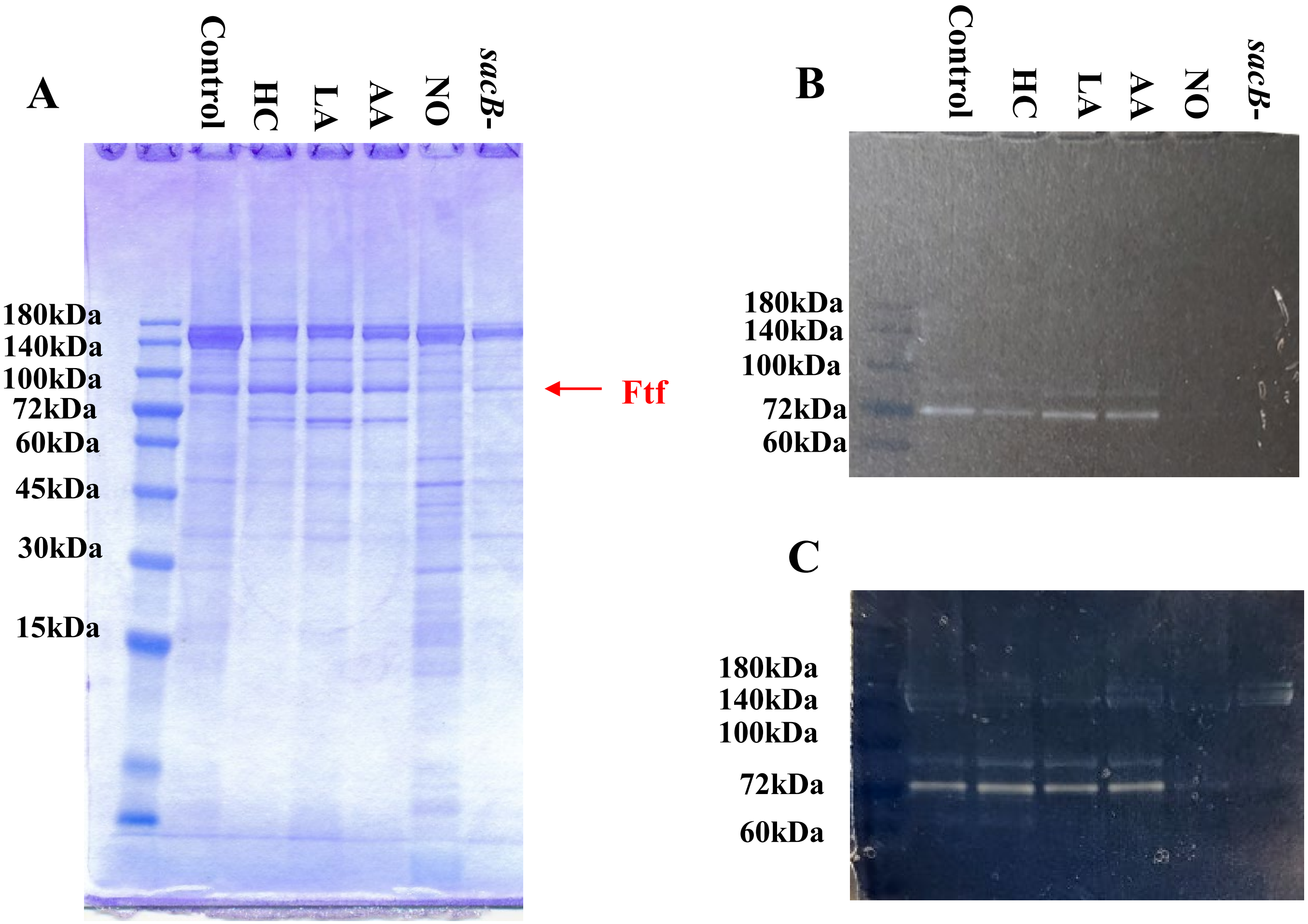

A
Control
HC
LA
AA
NO
sacB-
180kDa
140kDa
100kDa
72kDa
60kDa
45kDa
30kDa
15kDa
Ftf
B
Control
HC
LA
AA
NO
sacB-
180kDa
140kDa
100kDa
72kDa
60kDa
C
180kDa
140kDa
100kDa
72kDa
60kDa

Figure S7. Effects of initial pH condition on Ftf volume and activities in the MVs from *S. mutans*

Volume of Ftf on MVs from *S. mutans* UA159 extracted in control, HC, LA, AA, and NO, and *S. mutans* UA159 *sacB*$^{-}$ were analyzed in 12.5% polyacrylamide gel, SDS-PAGE and CBB staining (A), and zymography for overnight (B) and 3 days cultures using buffer involving raffinose (C). Representative data from more than three independent experiments are shown in the pictures.
